# A Compact 5.5 GHz Band-Rejected UWB Antenna Using Complementary Split Ring Resonators

**DOI:** 10.1155/2014/528489

**Published:** 2014-05-22

**Authors:** M. M. Islam, M. R. I. Faruque, M. T. Islam

**Affiliations:** ^1^Space Science Centre (ANGKASA), Universiti Kebangsaan Malaysia, 43600 Bangi, Selangor, Malaysia; ^2^Department of Electrical, Electronic and Systems Engineering, Universiti Kebangsaan Malaysia, 43600 Bangi, Selangor, Malaysia

## Abstract

A band-removal property employing microwave frequencies using complementary split ring resonators (CSRRs) is applied to design a compact UWB antenna wishing for the rejection of some frequency band, which is meanwhile exercised by the existing wireless applications. The reported antenna comprises optimization of a circular radiating patch, in which slotted complementary SRRs are implanted. It is printed on low dielectric FR4 substrate material fed by a partial ground plane and a microstrip line. Validated results exhibit that the reported antenna shows a wide bandwidth covering from 3.45 to more than 12 GHz, with a compact dimension of 22 × 26 mm^2^, and VSWR < 2, observing band elimination of 5.5 GHz WLAN band.

## 1. Introduction

The formation of the split ring resonator (SRR) was discovered by Pendry for the first time to construct metamaterials where the electromagnetic (EM) wave conducted in an opposite route according to the convectional manner [1–4]. The band-rejected properties are applied to extrude the undesired band for an ultrawide band antenna. In the field of short-distance wireless communication, a new opportunity is introduced by the Federal Communications Commission (FCC), with the announcement of 3.1–10.6 GHz frequency band for unlicensed radio communication [5]. Antennas include a spacious range of cellular mobile phones in the running society resulted in enhancing concerns combining its harmful radiation [6–8]. Because of having the opportunities of high data transmission rate, inexpensiveness, simplicity, and low spectral power density, UWB technology has been considered as one of the most fruitful candidates in wireless communications. Conventional antennas used parasitic strips, SRRs, and different slots to create rejected frequency bands. Some of these antennas had relatively large size or did not cover 5.5 GHz band rejection. Some UWB antennas are designed with band-notched, which are referred to below to face the effect created with respect to the frequency interference operating in WLAN (5.15–5.825 GHz) [9] as well as WiMAX (5.25–5.85 GHz) systems.

A new ring antenna fed by a microstrip line was proposed for UWB communication, where a proximity-coupled configuration was adopted [10]. The average gain was 2.93 dBi where the overall antenna dimension was 44 × 40 mm^2^. An UWB monopole antenna was presented with band-rejected features in the reported reference [11]. Two identical square complementary split ring resonators (CSRRs) were inserted on the radiating patch to achieve rejected bands. The antenna dimensions were 30 mm × 34 mm which was too large than our proposed antenna of compact size 22 mm × 26 mm. A miniaturized crescent microstrip antenna was proposed with an elliptical radiating patch by carving a circular hole inside symmetrically for UWB application [12]. The antenna had a relatively large size (45 × 50 mm^2^) and did not cover the upper edge frequency of the UWB. A tapered-shape slot antenna was proposed for ultrawide band applications [13]. For the radiating patch and the slot, different types of shapes such as triangular, rectangular, elliptical circular, and square were compared to each other. The antenna achieved ultrawide band properties with medium average peak gain of 3.6 dBi. Some antennas were proposed based on SRRs with metamaterials characteristics [14–16].

In [17], a monopole antenna was presented with single notched-band properties for UWB application. An open-looped resonator is inserted to originate notched-band centered at 5 GHz at the center of the reported fork-shaped antenna, where the antenna dimension is 35 × 30 mm^2^. A compact monopole antenna was presented with standard notched-band properties where the antenna dimensions were 30 mm × 35 mm [18]. In order to originate a notch frequency band covering from 5.12 to 6.08 GHz, a vertical coupling strip was inserted at the centre of the radiating slot patch. A notched-band design was presented for UWB antennas [19]. Complementary split ring resonators (CSSRs) were etched in the T-stub region of a CPW feed in the reported antenna to generate a notch frequency band of 5-6 GHz where the antenna dimensions were 33 mm × 35 mm.

A novel tapered slot antenna was proposed with a band-notched function for ultrawide band radios [20]. The reported antenna of 50 × 50 mm^2^ dimension attained ultrawide band characteristics with a band-notched of 4.6–6.2 GHz, implanting an Archimedean spiral-shaped slot into a microstrip-slot line feeding that belongs to open-circuit circular stub. In [21], genetic algorithm (GA) was applied to face various difficulties of microstrip antennas. In order to introduce and regulate notch bands, complex filter structures are required for some of the reported designs, in spite of having notched-band properties. On the other hand, the notched frequency bands of several reported antennas are more spacious than the desired band ranging from 5.15 to 5.825 GHz for WLAN applications, ending in downfall of holding lower signal quality and information.

An eliminated 5.5 GHz WLAN band microstrip antenna with slotted complementary SRRs that attains a compact UWB profile physically belonging to nearly omnidirectional radiation characteristics, gain, and reasonable current distribution is presented in this study. The mentioned band-rejected antenna is made of circular radiating patch with complementary SRRs slots and a partial ground plane containing a rectangular slot on the upper portion, generating an ultrawide bandwidth ranging from 3.45 to more than 12 GHz. The antenna formation is smooth with simple design and comfortable fabrication. Slotted CSRRs are inserted inside the circular patch to generate a notch frequency band for filtering out 5.5 GHz WLAN band. All the design parameters are related to create band rejection. The thickness and the split gap of the edges of the CSRR rings exhibit band-rejection UWB characteristic creating 5.5 GHz rejection band. By virtue of significant selection of slotted CSRRs parameters, it is observed that the reported antenna can obtain ultrawide band with rejected 5.5 GHz WLAN band.

## 2. Antenna Structures

**Figure 1:**
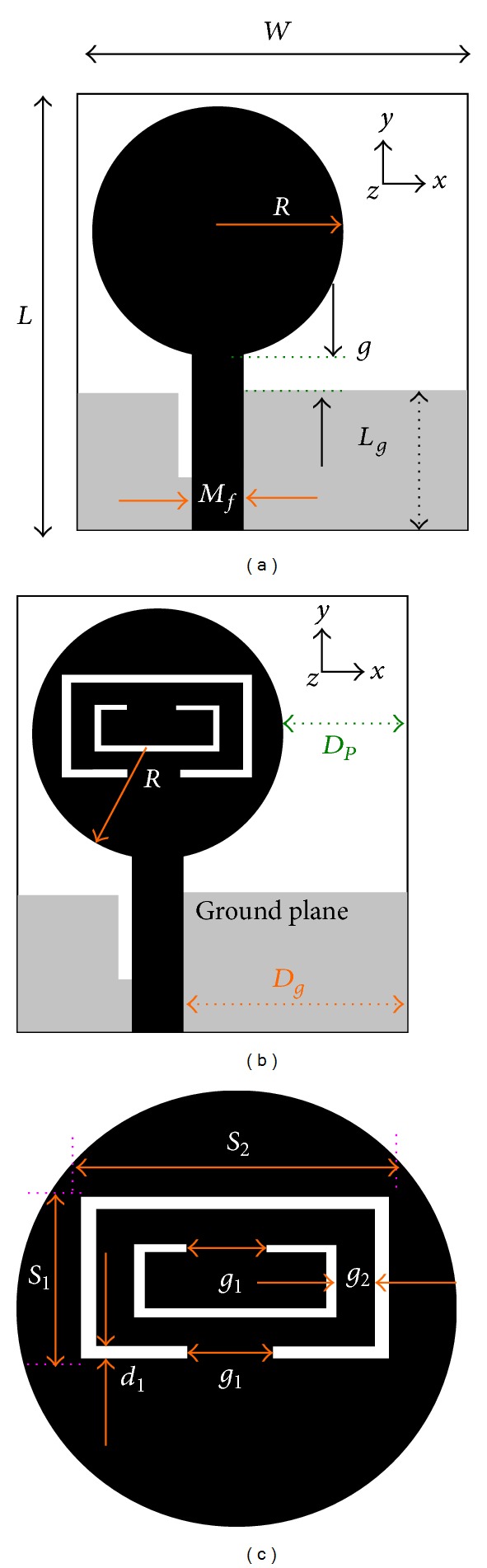
Structures of the (a) UWB antenna, (b) proposed band-rejected antenna, and (c) band-rejection layout.

Figure 1 illustrates the structures with detailed configurations of the proposed rejected 5.5 GHz WLAN band UWB antenna, which is printed on a low cost 1.6 mm thick FR4 substrate material belonging to dielectric constant of 4.6 and loss tangent of 0.02. All the simulations have been performed using commercially available software package, high frequency structural simulator (HFSS), which is based on finite element method (FEM). Depending on the UWB antenna indicated in Figure 1(a), the proposed antenna has been designed. It is made of a circular patch with a radius of *R* and a partial ground with a rectangular slot to acquire ultrawide bandwidth. The circular radiator is printed on the upper portion of the substrate fed by a microstrip line, where, on the lower portion of the substrate, the partial ground of *W* × *L*_*g*_ dimension is printed. The width at *M*_*f*_ and the length at 9.09 mm are constant in order to acquire 50 Ω input impedance. The port of the microstrip feed line is attached to a subminiature version A (SMA) connector. *g* is indicated as the gap between the ground plane and the radiating patch. *W* × *L* is the thorough size of the proposed antenna.

Slotted CSRRs are implanted at the middle position of the circular radiator to create a notched WLAN band centered at 5.5 GHz. The CSRRs are made of two rectangular split ring slices to implement the characteristics of metamaterials. The length of the rectangular slotted rings is smaller than a half wavelength at the resonant frequency. This leads the rejected band to be generated centered at 5.5 GHz WLAN band. However, the position of the CSRR plays an important role in order to determine the rejected 5.5 GHz WLAN frequency band of the proposed antenna. In case of UWB notched antenna structure, the function of the filter is to create and control rejected band sharply. Because of exhibiting this characteristic, this CSRR design is a filter structure. In spite of implanting slotted CSRRs, the thorough antenna size is constant where, for the filter structure, excessive space is no longer demanded.

**Figure 2:**
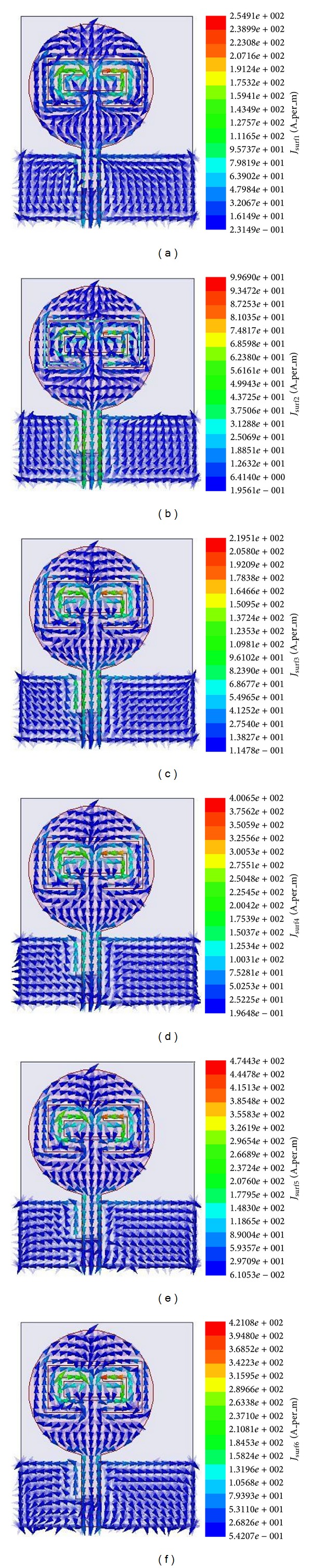
Surface current at 5.5 GHz for different values of the phase angle (a) 0°, (b) 30°, (c) 60°, (d) 90°, (e) 120°, and (f) 150°.

Figure 2 demonstrates the surface current with different values of the phase angle such as 0°, 30°, 60°, 90°, 120°, and 150° to acquire some acuteness of generating the rejected band centered at 5.5 GHz WLAN band. The surface current distribution of the antenna is acquired taking into account the optimized design parameters. It can be seen from Figure 2 that the concentration of the surface current distribution is very stable around the slotted split ring resonators and the feeding line. The flows of the current in the slotted CSRRs are inversed to the current flows in the outward edges of the radiator patch and the ground.

**Figure 3:**
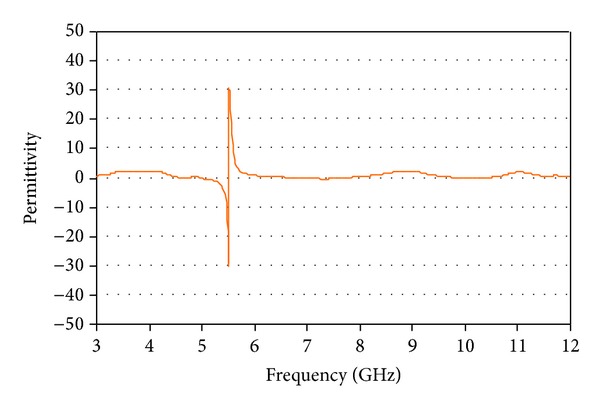
Extracted negative permittivity of CSRRs.

Consequently, the entire effective radiations are reduced enough or cancelled out completely, and an eliminated WLAN band is generated at 5.5 GHz by virtue of high attenuation. The extracted negative permittivity of the CSRRs is shown in Figure 3, which is clearly seen the single negative metamaterial characteristic. The single negative metamaterials characteristics operations denote epsilon negative (ENG), which means that the permittivity characteristic is negative.

## 3. Electromagnetic Performance Analysis

**Figure 4:**
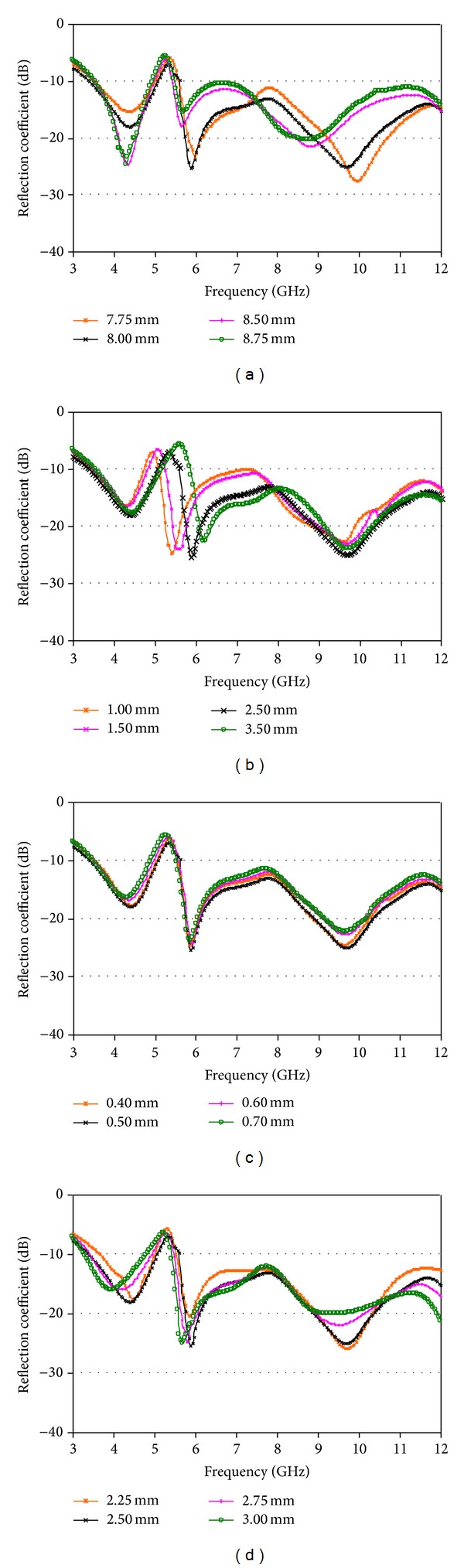
Effects for different values of (a) *R*, (b) *g*_1_, (c) *d*_1_, and (d) *M*_*f*_ on the simulated reflection coefficient.

The UWB antenna with removed 5.5 GHz WLAN band using slotted CSRRs has been discussed with the parametric analysis. The parametric analysis is executed in order to review the effects of design parameters, which are detailed as follows. All the parameters are retained constant in the simulation without the interested parameter. The radius of the circular patch for different values of *R* is indicated in Figure 4(a). The resonance of the eliminated band is found at 5.5 GHz using 8.00 mm as the value of *R*. If the value of *R* is increased or decreased, the resonance of the eliminated band is shifted sharply. Therefore, the optimized value of *R* is 8.00 mm.  Figure 4(b) demonstrates the gap among the two edges of the slotted rings for different values of *g*_1_. The resonance of the eliminated band switches from lower frequency up to a definite value, since the values of *g*_1_ rise. Hence, 2.5 mm is the optimized value.

Figure 4(c) depicts the impedance properties of the proposed antenna for different values of *d*_1_, which is the thickness of the CSRR. It is found that the resonance is on the lower frequency using 0.40 mm, the value of *d*_1_, while the resonance is observed on the higher frequency using 0.60 mm and 0.70 mm. The notched 5.5 GHz WLAN band is seen with 0.50 mm, which is desired. The impedance properties with different values of the feed line width, *M*_*f*_, are shown in Figure 4(d). The eliminated 5.5 GHz WLAN band is acquired, when the width of the feed line is 2.5 mm. Otherwise, the eliminated band is shifted on lower frequency or higher frequency using 2.25 mm, 2.75 mm, and 3.00 mm. It is realized from Figure 4 that the eliminated 5.5 GHz WLAN band is originated and regulated by virtue of slotted CSRRs. The optimized values of the designed parameters are as follows: *W* = 22 mm, *L* = 26 mm, *R* = 8 mm, *M*_*f*_ = 2.5 mm, *L*_*g*_ = 8 mm, *g* = 1.09 mm, *D*_*g*_ = 11.75 mm, *D*_*P*_ = 5 mm, *S*_1_ = 6.5 mm, *S*_2_ = 12.5, *g*_1_ = 2.5 mm, *g*_2_ = 1.5 mm, and *d*_1_ = 0.5 mm.

## 4. Experimental Verification

**Figure 5:**
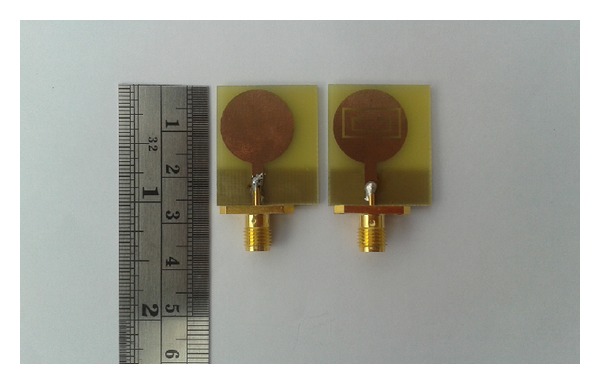
Photograph of the prototyped antenna.

**Figure 6:**
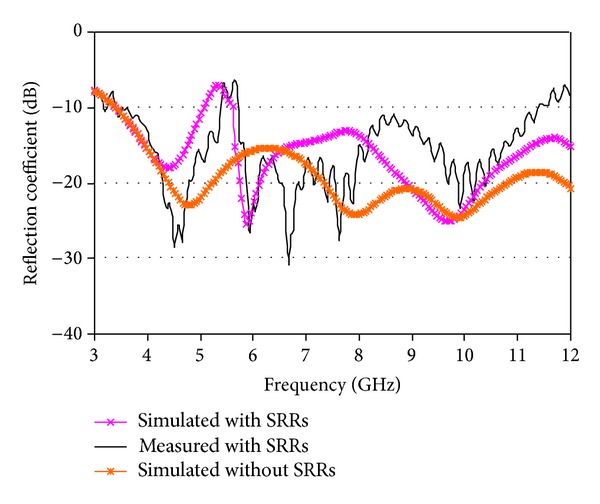
Measured and simulation reflection coefficient.

A vector network analyzer (model number: Agilent E8362C) has been used for the measurements in a standard far-field testing environment. The prototype of the proposed antenna is shown in Figure 5. The simulated and measured reflection coefficient of the mentioned antenna is illustrated in Figure 6 with reflection coefficient without SRRs. The eliminated 5.5 GHz WLAN band is realized in the reflection coefficient properties belonging to a bandwidth of 495 MHz. It covers an ultrawide frequency band from 3.45 GHz to more than 12 GHz with an eliminated 5.5 GHz WLAN band. The incompatibility between measurement and simulation is predominantly owing to fabrication faults in the antenna design.

**Figure 7:**
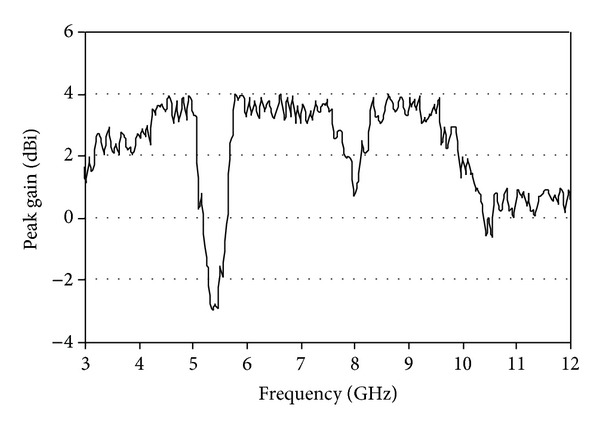
Measured peak gain of the proposed antenna.

Figure 7 indicates the measured peak gain of the reported antenna. The gain drops swiftly in the region of the eliminated 5.5 GHz WLAN band, as maximum radiated power is backed to the antenna at the notched band. The measured gain is −3 dBi in case of the eliminated band for WLAN. The antenna discloses stable gain without the operating frequency of the notched band.

**Figure 8:**
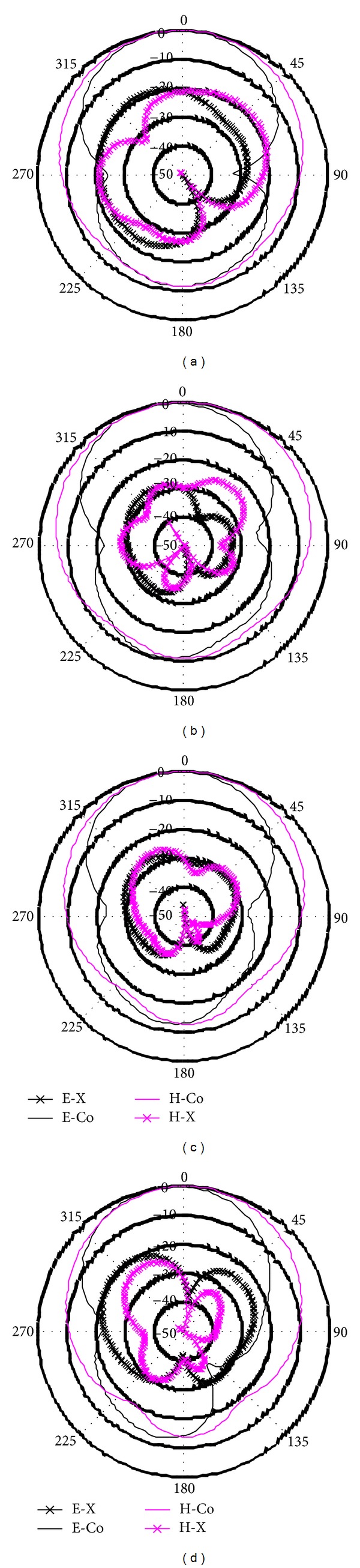
Measured radiation patterns at (a) 4.5, (b) 5.5, (c) 6.0, and (d) 6.7 GHz.

Figure 8 displays the measured radiation patterns at 4.5 GHz, 5.5 GHz, 6.0 GHz, and 6.7 GHz with E-plane and H-plane. In order to demonstrate copolarization and cross polarization, two-dimensional (2D) radiation patterns were resorted at resonances 4.5 GHz, 5.5 GHz, 6.0 GHz, and 6.7 GHz, respectively. *E*_*θ*_ represents the copolarization properties, and *E*_*φ*_ represents the cross polarization properties. The *yz* coordinates are taken into account as the E-plane and *xz* coordinates as the H-plane. The cross polarization dimension is smaller than the copolarization dimension on both the H-plane and E-plane at the resonances 4.5 GHz, 5.5 GHz, 6.0 GHz, and 6.7 GHz, respectively. It is observed that the proposed antenna exhibits better broadside radiation features and considerable front-to-back ratio with low cross polarization, which leads to symmetric and nearly omnidirectional radiation pattern. At the eliminated 5.5 GHz WLAN band, the gains are markedly restrained in all the directions, which announce the effect of slotted CSRRs.

**Figure 9:**
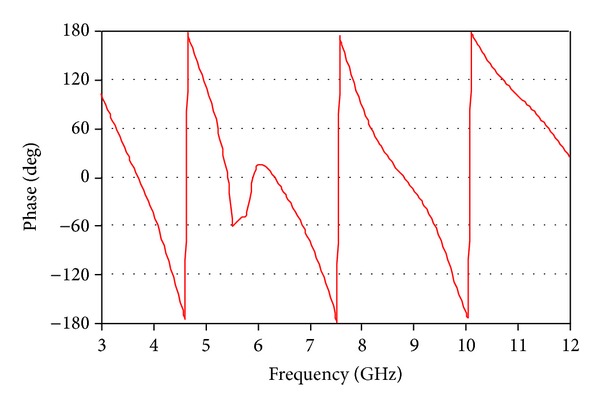
Measured phase variation of the input impedance.

The measured phase variation of the input impedance is shown in Figure 9. It is overlooked that the phase variation is linear over the entire operating frequency bands without the eliminated 5.5 GHz WLAN band.

**Figure 10:**
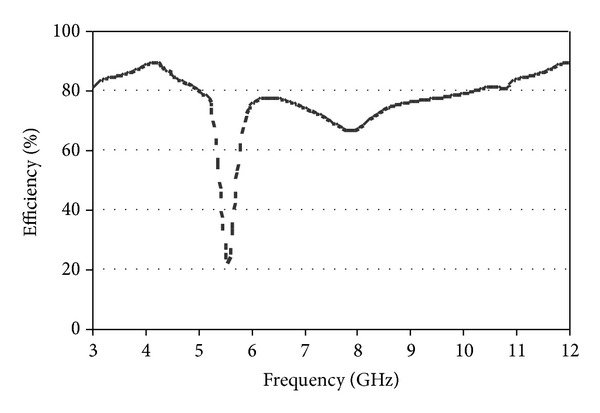
Radiation efficiency of the proposed antenna.

The antenna radiation efficiency is shown in Figure 10. From Figure 10, it can be found that the antenna acquired good radiation efficiency throughout the entire UWB range without 5.5 GHz rejected band. The efficiency reduces sharply at 5.5 GHz band that illustrates the effect of rejected band. The comparisons between existing and proposed band-rejected UWB antennas are listed in Table 1, which bears testimony to the compact profile of the proposed antenna.

## 5. Conclusion

A compact UWB antenna with rejected 5.5 GHz WLAN band using complementary split ring resonators (CSRRs) has been presented and studied in this paper. The antenna is composed of a circular patch and a partial ground with a thorough size of 22 × 26 mm^2^. The slotted CSRRs are inserted in the circular patch to observe notched 5.5 GHz WLAN band characteristics. The proposed antenna acquired better UWB execution with notched band properties to lighten the interference problem created from WLAN. The properties of wide bandwidth, nearly omnidirectional radiation patterns, and compact profile satisfactory gain with rejection of rejected 5.5 GHz WLAN band originates the proposed antenna as a milestone for UWB applications.

## Figures and Tables

**Table 1:** Comparisons between existing and proposed band-rejected UWB antennas.

Antennas	Rejected band (GHz)	Dimensions (mm^2^)
[18]	5.12–6.08	30 × 35
[22]	4.85–6.04	30 × 36
[23]	5.15–5.825	30 × 39.3
[17]	Centre at 5.00	35 × 30
[24]	5.00–6.00	35 × 35
Proposed antenna	5.41–5.72 (Centre at 5.5)	22 × 26
